# Anatomical Basis of Tetanus

**Published:** 1882-11

**Authors:** Fr. Schultze


### ANATOMICAL BASIS OF TETANUS.

Investigations made in four cases of traumatic tetanus failed to show anything in the central nervous system that would speak for myelitis or meningitis. For this reason it is argued there is little confidence to be placed in some vague reports, recounting various pathological phenomena found in cases of traumatic and idiopathic tetanus.—*Fr. Schultze, Neurolog. Cbl.*, 1882, No. 6.

